# Anti-Inflammatory Effect of Oleuropein in Experimental Rat Spinal Cord Trauma

**Published:** 2012-04-01

**Authors:** A R Khalatbary, Gh R Zarrinjoei

**Affiliations:** 1 Department of Anatomy, Faculty of Medicine, Mazandaran University of Medical Sciences, Khazar Boulevard, Sari, Iran; 2 Razi Herbal Medicines Research Center, Lorestan University of Medical Sciences, Khorramabad, Iran

**Keywords:** Oleuropein, Spinal cord, Trauma, Inflammation, Rat

## Abstract

**Background:**

Spinal cord injury stimulates an inflammatory reaction that causes substantial secondary damage inside the injured spinal tissue. The purpose of this study was to determine the anti-inflammatory effect of oleuropein on traumatized spinal cord.

**Methods:**

Rats were randomly divided into four groups of 7 rats each as follows: Sham-operated group, trauma group, and oleuropein treatment groups (20 mg/kg, ip, immediately and 1 hour after spinal cord injury). Spinal cord samples were taken 24 hours after injury and studied for immunohistochemistry of tumor necrosis factor-α (TNF-α), interleukin-1ß (IL-1ß), nitrotyrosine, inducible nitricoxide synthase (iNOS), cyclooxygenase-2 (COX-2), and poly(ADP-ribose) polymerase (PARP).

**Results:**

Attenuated TNF-α, IL-1ß, nitrotyrosine, iNOS, COX-2, and PARP expression could be detected in the oleuropein-treated rats.

**Conclusion:**

Oleuropein modulates inflammatory reactions following spinal cord injury.

## Introduction

Neurological damages after traumatic spinal cord injury result from both primary mechanical injury and secondary degeneration process. Outcome of spinal cord injury depends on the extent of secondary damage mediated by a series of cellular, molecular and biochemical cascades, including calcium ion influx, oxygen free radical-induced lipid peroxidation, inflammatory reaction, autoimmune response, vascular events, and apoptosis.[1][2][3] In recent years, much attentions have been focused on secondary injury because it appears to be susceptible to therapeutic interventions that may include using of free radical scavengers and anti-inflammatory agents. Olive oil is a rich source of phenolic components such as oleuropein, which has many beneficial health effects in human.[4] On the other hand, hydrolysis of oleuropein results in the formation of other phenolicis, including hydroxytyrosol and tyrosol.[5] Experimental studies attributed the beneficial effects of oleuropein and its derivatives such as hydroxytyrosole, to a variety of biological activities, including free radical scavenging/antioxidant, anti-inflammatory, anti-carcinogenic, anti-microbial, anti-atherogenic, and antiviral properties.[6][7][8] Some studies documented that oleuropein elicits anti-inflammatory effects by lypoxygenase activity and the production of leukotriene B4,[9] inhibiting biosynthesis of proinflammatory cytokines[10][11] or modulating inflammatory parameters.[12] Olive oil phenols have been shown to have some of protective effects against brain hypoxia-reoxigenation,[13][14] cerebral ischemia,[15][16] brain damage after hypoxiareoxygenation in diabetic rats,[17] and ageing.[18] Although the exact neuroprotective mechanism of olive oil phenols is unclear, the antioxidative and anti-inflammatory effects of these phenols are considered to be the main mechanisms leading to this neuroprotective effect.

In the present study, we investigated the potential anti-inflammatory effect of oleuropien immunohistochemically in rat as an experimental model of spinal cord trauma.

## Materials and Methods

Male adult Spargue-Dawley rats were used (250-300 g, Pasteur?s Institute, Tehran, Iran) in this study. They were kept under standard conditions according to the guidelines of the university?s animal care codes to minimize the animal?s suffering. The study protocol was approved by the Research and Ethics Committee of Lorestan University of Medical Sciences.

Contusive spinal cord injury was carried out using the weigh dropping technique. The animals were anesthetized with ketamine (75 mg/kg, ip) and xylazine (10 mg/kg, ip). Laminectomy was performed at T9 level vertebra; the dorsal surface of the cord was then subjected to weight drop impact using a 10-g weight dropped from a height of 2.5 cm in order to produce contusive spinal cord injury. Following the surgery, the recovery of the animals was assisted by administering lactated ringer's solution (12-25 ml) subcutaneously immediately after surgery and cefazolin (50 µg/kg, Jaber Ibn Hayan, Tehran, Iran) which was administered twice daily for 3 days. The urinary bladder was pressed three times a day until the function was retained.

The rats were randomly allocated into four groups, each containing 7 rats: (i) sham- operated group, which underwent laminectomy alone; (ii) trauma group, which underwent laminectomy followed by spinal cord injury and received saline (vehicle); (iii and iv) OE treatment groups, which underwent laminectomy followed by spinal cord injury and received a 20- mg/kg single dose of oleuropien (purchased from Sigma-Aldrich) intraperiteonally immediately (oleuropien 1) and 1 hour (oleuropien 2) after trauma, respectively.

At 24 hours after spinal cord injury,[19] the spinal cords, which contained the contusion epicenter, were fixed in 10% (wt/vol) PBS-buffered formaldehyde and embedded in paraffin. Eight-micrometer sections were serially cut horizontally from each block. For immunohistochemistry, sections were incubated in normal serum (in order to block non-specific site), and anti-iNOS rabbit polyclonal antibody (1:50 in PBS, vol/vol, Abcam), anti-COX-2 rabbit polyclonal antibody (1:100 in PBS, vol/vol, Abcam), anti-PARP rabbit polyclonal antibody (1:100 in PBS, vol/vol, Abcam), anti-nitrotyrosine rabbit polyclonal antibody (1:50 in PBS, vol/vol, Millipore), Biotin antimouse/rat IL-1ß antibody (10 µg/ml in PBS, w/vol, Biolegend), or anti-rat TNF-α antibody (5 µg/ml in PBS, w/vol, R&D) overnight at 4°C. Sections were washed with PBS and then incubated with ultratek HRP (anti-polyvalent) (ScyTek), streptavidin-HRP (Millipore), or HRP conjugated rabbit anti-goat secondary antibody (Millipore) and demonstrated with diaminobenzidine tetrahydrochloride for 10 minutes. Afterwards, they were counterstained with hematoxylin, dehydrated, and mounted. For negative controls, primary antibodies were omitted. For quantitative analysis, immunohistochemical photographs (n=5, photos from each samples collected from all rats in each experimental group) were assessed by densitometry using MacBiophotonics Image J 1.41a software on an ASUS personal computer.

Statistical analysis was carried out using the SPSS package (Version 15, Chicago, IL, USA). Results were presented as mean values (±SD). The K-S test was used in order to evaluate the normality of the data. Also, the Tukey's multiple comparison test and the analysis of the variance were used in order to compare each two groups and compare the data among the groups, respectively. A value of p<0.05 was considered significant.

## Results

**Fig. 1:**
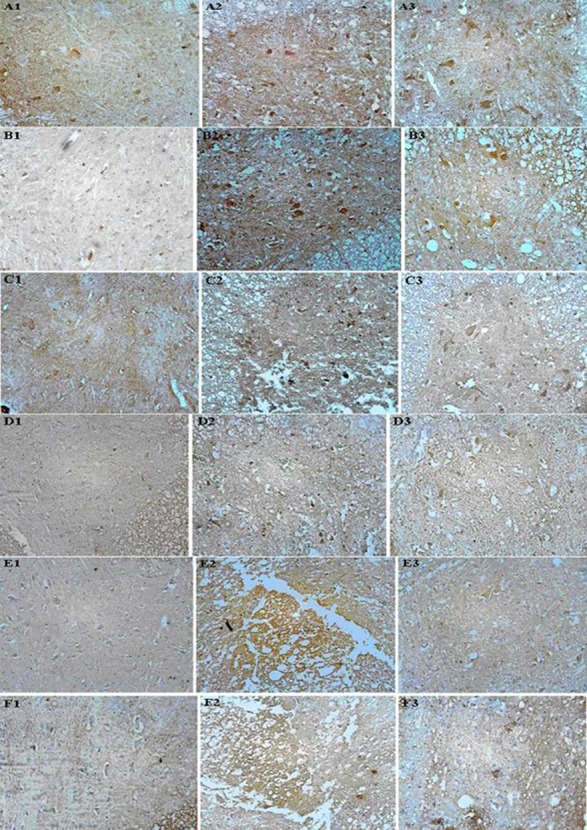
Immunohistochemical expression of COX-2, iNOS, nitrotyrosine, PARP, TNF-α, and IL-1β. Light photomicrographs show COX-2 reactivity (A1=sham; A2=trauma; A3=OE treatment), iNOS reactivity (B1=sham; B2=trauma; B3=OE treatment), nitrotyrosine reactivity (C1=sham; C2=trauma; C3=OE treatment), PARP reactivity (D1=sham; D2=trauma; D3=OE treatment), TNF-α reactivity (E1=sham; E2=trauma; E3=OE treatment), and IL-1β reactivity (F1=sham; F2=trauma; F3=OE treatment) 24 hours after injury (magnification, ×200). The positive staining of the antigens is presented by a brown color.

Figure 1 shows the immunohistochemical staining of COX-2 (A), iNOS (B), nitrotyrosine (C), PARP (D), TNF-a (E), and IL-1ß (F), respectively. Almost no positive reaction could be detected in sham-operated groups for all of the antigens (A1, B1, C1, D1, E1 and F1), whereas the spinal cord sections of traumatized rats exhibited an increased positive staining for these antigens (A2, B2, C2, D2, E2 and F2). Oleuropien treatment reduced significantly the degree of positive staining for all of the antigens (A3, B3, C3, D3, E3 and F3). Densitometry analysis of the photographs showed that immunohistochemical expression of COX-2 in the oleuropien 1 (0.4±0.02) and the oleuropien 2 (0.26±0.06), iNOS in the oleuropien 1 (0.53±0.17) and the oleuropien 2 (0.35±0.07), nitrotyrosine in the oleuropien 1(0.47±0.26) and the oleuropien 2 (0.23±0.06), PARP in the oleuropien 1 (0.25±0.12) and the oleuropien 2 (0.23±0.02), TNF-a in the oleuropien 1 (0.35±0.05) and the oleuropien 2 (0.39±0.0), IL-1ß in the oleuropien 1 (0.67±0.04) and the oleuropien 2 (0.57±0.19) groups were significantly (p<0.01) lower than their trauma groups (1.43±0.08, 1.51±0.27, 1.3±0.47, 0.73±0.17, 0.83±0.03, and 1.96±0.11, respectively). Meanwhile, the differences between the oleuropien 1 and the oleuropien 2 groups for all of the antigens were not significant (p>0.05). On the other hand, the differences between the trauma groups and their sham groups (0.23±0.1, 0.09±0.01, 0.13±0.04, 0.07±0.0, 0.07±0.02, and 0.15±0.05, respectively) were significant (p<0.001).

## Discussion

Secondary auto-destructive processes of spinal cord injury have a highly debilitating pathology, considered to be a number of interrelated processes. Injury to the spinal cord provokes a local inflammatory response which amplifies the secondary damage. The inflammatory response involves non-cellular and cellular components. It is well documented that the potent pro-inflammatory cytokines, including tumor necrosis factor alpha (TNF-α) and interleukin-1 beta (IL-1ß), which are synthesized immediately after injury, playing detrimental roles in post-traumatic injury associated with spinal cord injury.[20] TNF-α and IL-1ß are involved in a wide range of events, including vascular permeability,[21] recruitment of inflammatory cells,[22] induction of inducible nitric oxide synthase (iNOS)[23] and cyclooxigenase-2 (COX-2)[24] in the injury site. In this regard, it has been well demonstrated that the blocking of TNF-α or IL-1ß confers neuroprotection and improves functional recovery following experimental spinal cord injury.[25] On the other hand, TNF-α and IL-1ß play a central role in the induction of iNOS.[21] iNOs is a one of the three distinct enzymes that produces nitric oxide (NO), a free radical gas molecule which is known to have a crucial role in the development of the secondary inflammatory response and apoptosis following traumatic spinal cord injury.[26] In this regard, some studies have clearly demonstrated that attenuation of iNOS expression is secondary to a reduced formation of endogenous TNF-α and IL-1ß.[25] Similar to iNOS, the expression of COX-2, a enzyme which is involved in the generation of some inflammatory mediators, is also mediated by TNF-α and IL-1ß.[24] Cyclooxygenase inhibitors can improve functional outcome after spinal cord contusion.[27] In this study, we demonstrated that oleuropein treatment attenuated significantly expression of TNF-α and IL-1ß, and consequently expression of iNOS and COX-2. Although the most famous and widely renowned properties of olive phenolics have long been attributed to the antioxidant and free radical scavenging effects,[6] however, emerging evidences have shown the anti-inflammatory effects of these phenolics.[8][28][29] It has been well established that olive phenolics inhibit iNOS activity or the inflammatory mediators that stimulate this enzyme following brain hypoxia-reoxygenation.[13] Olive oil phenolic compounds decrease the circulating concentrations of IL-6,[30] a pro-inflammatory agent that stimulates inflammation in response to trauma. Another study has also shown that the olive oil phenolic compounds inhibit COX-2 activity.[31] Impellizzeri et al.,[11] reported that administration of oleuropein in a mouse model of carrageenan-induced pleurisy, caused a significant reduction of TNF-α, IL-1ß and NO.

Peroxynitrite, a cytotoxic molecule produced in the spinal cord tissue following trauma, contributes to the post-traumatic inflammatory reaction including tyrosine nitration and lipid peroxidation,[32] and also cause DNA damage resulting in the activation of poly (ADP-ribose) polymerase (PARP).[33] On the other hand, overactivation of PARP, a nuclear enzyme which is activated by strand break in DNA, results in depletion of NAD and ATP and ultimately cell death.[34] It has been clearly demonstrated that spinal cord injury induced PARP activation, and treatment with PARP inhibitors significantly reduced the development of inflammation and apoptosis in the traumatized tissue.[35] In present study, we demonstrated that oleuropein treatment attenuated significantly expression of PARP and nitrotyrosine. In this regard, some investigations have shown that olive oil polyphenols significantly reduced peroxynitrite formation.[36] Another study documented that administration of oleuropein attenuates nitrotyrosine and PARP.[11]

Finally, our results showed that administration of oleuropein immediately and 1 hour after spinal cord injury, significantly attenuated inflammatory responses.

## References

[1] Amar AP, Levy ML (1999). Pathogenesis and pharmacological strategies for mitigating secondary damage in acute spinal cord injury.. Neurosurgery.

[2] Jazayeri-Shooshtari SM, Namdar Z, Owji SM, Mehrabani D, Mohammadi-Samani S, Tanideh N, Alizadeh AA, Namazi H, Amanollahi A, Rajaee Z, Bidaki L (2009). Healing effect of lamotrigine on repair of damaged sciatic nerve in rabbit.. J Appl Anim Res.

[3] Rakei SM, Rahmanian A, Safarian A, Azarpira N, Mehrabani D (2009). The effect of bioglue on cerebral cortex in experimental rats.. Iran Red Crescent Med J.

[4] Waterman E, Lockwood B (2007). Active components and clinical applications of olive oil.. Altern Med Rev.

[5] Visioli F, Bernardini E (2011). Extra virgin olive oil's polyphenols: biological activities.. Curr Pharm Des.

[6] Visioli F, Poli A, Gall C (2002). Antioxidant and other biological activities of phenols from olives and olive oil.. Med Res Rev.

[7] Cicerale S, Lucas L, Keast R (2010). Biological activities of phenolic compounds present in virgin olive oil.. Int J Mol Sci.

[8] Omar SH (2010). Oleuropein in olive and its pharmacological effects.. Sci Pharm.

[9] de la Puetra R, Ruiz Gutierrez V, Hoult JR (1999). Inhibition of leukocyte 5-lipoxygenase by phenolics from virgin olive oil.. Biochem Pharmacol.

[10] Giamarellos-Bourboulis EJ, Geladopoulos T, Chrisofos M, Koutoukas P, Vassiliadis J, Alexandrou I, Tsaganos T, Sabracos L, Karagianni V, Pelekanou E, Tzepi I, Kranidioti H, Koussoulas V, Giamarellou H (2006). Oleuropein: a novel immunomodulator conferring prolonged survival in experimental sepsis by pseudomonas aeruginosa.. Shock.

[11] Impellizzeri D, Esposito E, Mazzon E, Paterniti I, Di Paola R, Bramanti P, Morittu VM, Procopio A, Britti D, Cuzzocrea S (2011). The effects of oleuropein aglycone, an olive oil compound, in a mouse model of carrageenan-induced pleurisy.. Clin Nutr.

[12] Puel C, Mathey J, Agalias A, Kati-Coulibaly S, Mardon J, Obled C, Davicco MJ, Lebecque P, Horcajada MN, Skaltsounis AL, Coxam V (2006). Dose-response study of effect of oleuropein, an olive oil polyphenol, in an ovariectomy/inflammation experimental model of bone loss in the rat.. Clin Nutr.

[13] Gonzalez-Correa JA, Munoz-Marin J, Arrebola MM, Guerrero A, Narbona F, Lopez-Villodres JA, De La Cruz JP (2007). Dietary virgin olive oil reduces oxidative stress and cellular damage in rat brain slices subjected to hypoxia-reoxigenation.. Lipids.

[14] Gonzalez-Correa JA, Navas MD, Lopez-Villodres JA, Trujillo M, Espartero JL, De La Cruz JP (2008). Neuro-protective effect of hydroxytyrosol and hydroxytyrosol acetate in rat brain slices subjected to hypoxia-reoxygenation.. Neurosci Lett.

[15] Bu Y, Rho S, Kim J, Kim MY, Lee DH, Kim SY, Choi H, Kim H (2007). Neuro-protective effect of tyrosol on transient focal cerebral ischemia in rats.. Neurosci Lett.

[16] Mohagheghi F, Bigdeli MR, Rasoulian B, Zeinanloo AA, Khoshbaten A (2010). Dietary virgin olive oil reduces blood brain barrier permeability, brain edema, and brain injury in rats subjected to ischemia-reperfusion.. ScientificWorldJournal.

[17] De La Cruz JP, Del Rio S, Arrebola MM, Lopez-Villodres JA, Jebrouni N, Gonzalez-Correa JA (2010). Effect of virgin olive oil plus acetylsalicylic acid on brain slices damage after hypoxia-reoxygenation in rats with type 1-like diabetes mellitus.. Neurosci Lett.

[18] Pitozzi V, Jacomelli M, Zaid M, Luceri C, Bigagli E, Lodovici M, Ghelardini C, Vivoli E, Norcini M, Gianfriddo M, Esposto S, Servili M, Morozzi G, Baldi E, Bucherelli C, Dolara P, Giovannelli L (2010). Effects of dietary extra-virgin olive oil on behaviour and brain biochemical parameters in ageing rats.. Br J Nutr.

[19] Hausmann ON (2003). Post-traumatic inflammation following spinal cord injury.. Spinal Cord.

[20] Hayashi M, Ueyama T, Nemoto K, Tamaki T, Senba E (2000). Sequential mRNA expression for immediate early genes, cytokines and neurotrophins in spinal cord injury.. J Neurotrauma.

[21] Schnell L, Fearn S, Schwab ME, Perry VH, Anthony DC (1999). Cytokine-induced acute inflammation in the brain and spinal cord.. J Neuropathol Exp Neurol.

[22] Pineau I, Lacroix S (2007). Proinflammatory cytokine synthesis in the injured mouse spinal cord: multiphasic expression pattern and identification of the cell types involved.. J Comp Neurol.

[23] Matsuyama Y, Sato K, Kamiya M, Yano J, Ivata H, Isobe K (1998). Nitric oxide: a possible etiologic factor in spinal cord cavitation.. J Spinal Disord.

[24] Tonai T, Taketani Y, Ueda N, Nishisho T, Ohmoto Y, Sakata Y, Muraguchi M, Wada K, Yamamoto S (1999). Possible involvement of interleukin-1 in cyclooxygenase-2 induction after spinal cord injury in rats.. J Neurochem.

[25] Genovese T, Mazzon E, Crisafulli C, Di Paola R, Muia C, Bramanti P, Cuzzocrea S (2006). Immunomodulatory effects of etanercept in an experimental model of spinal cord injury.. J Pharmacol Exp Ther.

[26] Genovese T, Mazzon E, Mariotto S, Menegazzi M, Cardali S, Conti A, Suzuki H, Bramanti P, Cuzzocrea S (2006). Modulation of nitric oxide homeostasis in a mouse model of spinal cord injury.. J Neurosurg Spine.

[27] Resnick DK, Graham SH, Dixon CE, Marion DW (1998). Role of cyclooxygenase 2 in acute spinal cord injury.. J Neurotrauma.

[28] de la Puerta R, Martinez-Dominguez E, Ruiz-Gutierrez V (2000). Effect of minor components of virgin olive oil on topical antiinflammatory assays.. Z Naturforsch C.

[29] Martinez-Domingues E, de la Puerta R, Ruiz-Gutierrez V (2001). Protective effects upon experimental inflammation models of a polyphenol supplemented virgin olive oil diet.. Inflamm Res.

[30] Fito M, Cladellas M, de la Torre R, Marti J, Munoz D, Schroder H, Al-cantara M, Pujadas-Bastardes M, Marrugat J, Lopez-Sabater MC, Bruguera J, Covas MI; SOLOS Investigators (2008). Anti-inflammatory effects of virgin olive oil in stable coronary disease patients: a randomized, crossover, controlled trial.. Eur J Clin Nutr.

[31] Beauchamp GK, Keas RS, Morel D, Lin J, Pika J, Han Q, Lee CH, Smith AB, Breslin PA (2005). Phytochemistry: ibuprofen-like activity in extra-virgin olive oil.. Nature.

[32] Blanchard-Fillion B, Souza JM, Friel T, Jiang GC, Vrana K, Sharov V, Barron L, Schoneich C, Quijano C, Alvarez B, Radi R, Przedborski S, Fernando GS, Horwitz H, Ischiro-poulos H (2001). Nitration and inactivation of tyrosine hydroxylase by peroxynitrite.. J Biol Chem.

[33] Ahmad R, Rasheed Z (2009). Biochemical and cellular toxicology of peroxynitrite: implications in cell death and autoimmune phenomenon.. Immunopharmacol Immunotoxicol.

[34] Scott GS, Szabo C, Hooper DC (2004). Poly(ADP-ribose) polymerase activity contributes to peroxynitrite-induced spinal cord neuronal cell death in vitro.. J Neurotrauma.

[35] Genovese T, Mazzon E, Muia C, Patel NSA, Threadgill MD, Bramanti P, De Sarro A, Thiemermann C, Cuzzocrea S (2005). Inhibitors of poly(ADP-ribose) polymerase modulate signal transduction pathways and secondary damage in experimental spinal cord trauma.. J Pharmacol Exp Ther.

[36] Deiana M, Aruoma OI, Bianchi ML, Spencer JP, Kaur H, Halliwell B, Aeschbach R, Banni S, Dessi MA, Corongiu FP (1999). Inhibition of peroxynitrite dependent DNA base modification and tyrosine nitration by the extra virgin olive oil-derived antioxidant hydroxytyrosol.. Free Radic Biol Med.

